# Rethinking love, independence, and speciesism in assistance dog discourse

**DOI:** 10.3389/fsoc.2024.1448676

**Published:** 2025-01-03

**Authors:** Birkan Taş

**Affiliations:** Section Sociology of Social Differentiation and Socio-culture, Department of Social Work and Social Welfare, Faculty of Human Sciences, University of Kassel, Kassel, Germany

**Keywords:** assistance dogs, love, affective labor, independence, interdependence, speciesism

## Abstract

This paper challenges the prevailing belief that assistance dogs inherently love their roles, arguing that the notion of “unconditional love” in discourses on assistance dog perpetuates a human-centric perspective and reinforces speciesism. It emphasizes the importance of recognizing the affective experiences of these working animals and of acknowledging the interdependence between people with disabilities and assistance dogs. The paper has four main objectives: (1) critiquing the concept of unconditional love attributed to assistance dogs, (2) recognizing the physical and affective labor of assistance dogs, (3) highlighting the importance of interdependence over independence, and (4) exploring the intersections of ableism and speciesism in the context of assistance dogs. By examining the role of love as a narrative-framing device, the paper aims to reveal how anthropocentric viewpoints often obscure the exploitation of assistance dogs. Incorporating insights from human-animal studies and disability studies, the paper seeks to enrich sociological research on emotions and power structures, advocating for a shift toward valuing the labor and wellbeing of assistance dogs. This approach challenges the liberal ideology of independence and promotes a more inclusive understanding of interspecies relationships, ultimately enhancing the sociological study of emotions, and intersections between sociology, disability studies, and human-animal studies.

## 1 Introduction

Assistance dogs provide support for people with disabilities by performing various tasks. They are often purpose-bred by accredited organizations to ensure that they possess the ideal temperament and physical traits tailored to their human companions’ needs (Bolak, 2024). There is a wide range of assistance dogs available, each trained to meet the specific needs of people with disabilities (Bremhorst et al., 2018). Examples include “guide dogs” helping blind people, and “hearing dogs” providing support for deaf people. All other assistance dogs are categorized as “service dogs” (Assistance Dogs International, 2024). Among them are “mobility assistance dogs” for people with balance issues, “medical alert” or “seizure dogs” for detecting hormonal changes in humans and alerting them, “psychiatric assistance dogs” for helping people deal with depression, anxiety, or stress disorders, and “autism assistance dogs” primarily helping children on the autism spectrum (Assistance Dogs International, 2024; Gross, 2006). Their job requires “real-time predictive or responsive responses, and round-the-clock involvement in serving someone’s needs” (Coulter, 2016, p. 59). Most assistance dogs begin their journey in accredited schools, where they are placed with volunteer foster families, known as puppy-raisers, to undergo basic obedience training focused on positive reinforcement rather than punitive methods like shock collars (Assistance Dogs International, 2024). Once the dogs—mostly Golden Retrievers and Labrador Retrievers—reach approximately one and a half years of age, they receive public conduct and distraction training at specialized assistance dog schools, which distinguishes them from most companion animals or emotional support dogs (Walther et al., 2017).

The physical and affective care work that assistance dogs perform is rooted in selective breeding of the most obedient dogs coupled with hundreds of hours of work and advance training (Price, 2017). Most assistance dogs start their lives in confined spaces, where they are conditioned to follow specific norms and commands from puppyhood. Behavioral conditioning often relies on food, and dogs showing fear or anxiety typically do not qualify as effective assistance animals (Tomkins et al., 2011). This paper challenges the common assumption that assistance dogs enjoy their roles and feel unconditional love for the humans they assist, calling for a deeper exploration of the implications of these beliefs. Such unquestioned presuppositions often conceal the realities of control, restriction, and the exploitation of canine labor. Instead, the paper argues that the work of assistance dogs should be understood as affective labor, with their wellbeing as a key focus. Scholarly discussions continue about the ethics of employing animals for ongoing service and caregiving roles, with some raising concerns that such practices could infringe upon the animal’s wellbeing, social relationships, and autonomy (Coulter, 2016). Given these considerations, one must question whether dogs genuinely enjoy their work or if they lack sufficient agency. That is why focusing on human-canine interaction, critiquing the notion of love, and emphasizing the relational dimension of such interactions can offer valuable insights for sociological research, particularly when applied to contexts such as critiques of anthropocentrism, which have received less attention in studies of love within the Sociology of Emotions.

When it comes to discussions on human-assistance dog interaction, human mental health, welfare, and quality of life comes first (Shintani et al., 2010; Rodriguez et al., 2021). Several studies have found that people who spend time with dogs experience reduced stress, anxiety, and social isolation (Friedmann and Son, 2009). In such human-centric perspectives, canine welfare or health is of secondary importance. This explains the limited amount of research conducted on the welfare of assistance dogs, as well as their behavioral and cognitive abilities (Bremhorst et al., 2018). For instance, existing research on the use of autism assistance dogs is said to be inconsistent, scarce, and human-centric (Harrison and Zane, 2017; Tseng, 2023). While some studies, such as Shintani et al. (2010), suggest that evidence for the positive impact of assistance dogs on human psychosocial health and wellbeing may be methodologically limited, this gap highlights the need to equally prioritize rigorous research into the welfare of assistance dogs themselves. Neglecting to understand and address physical and psychological welfare concerns in dogs poses risks not only to the dogs but also to people with disabilities and their caregivers (Burrows et al., 2008). For instance, autism assistance dogs often wear tether harnesses that prevent children from wandering, which can strain the dog physically and psychologically, compromising the dog’s wellbeing. If the dog becomes stressed or injured, this could lead to a breakdown in the caregiving dynamic, ultimately impacting the safety and support for both the child and the caregivers who rely on the dog’s assistance. This article emphasizes the significance and political urgency of reflecting on canine affects within the context of assistance dogs. The political urgency stems from the increasing reliance on assistance dogs in public and private sectors, coupled with growing advocacy for animal welfare rights, which demands immediate policy attention to ensure that both the animals and individuals with disabilities receive appropriate protection and care. This perspective underscores the article’s argument that the responsibility of caring for assistance dogs and individuals with disabilities is not separate but rather interconnected.

The common assumption prevalent in most assistance dog discourse is that dogs love working for people and provide increased independence for them (Oliver, 2016). Reduced to their functionality and performance, dogs are to find joy and fulfilment in their roles, deriving satisfaction from pleasing their disabled companions. Nevertheless, this particular viewpoint predominantly originates from liberal and anthropocentric perspectives, which reduce “everything to usable equipment or productive labor” and value human lives over nonhuman animals’ (Oliver, 2016, p. 247). Although dog trainers and handlers who state that assistance dogs love working and helping people are quite common (Cochrane, 2020), there are also a considerable number of scholars and animal activists who examine the issue through the lens of domination and exploitation (Sorenson, 2014; Taylor, 2017). This paper calls for a critical examination of the assumptions of unconditional love in assistance dog discourses, which play a pivotal role in shaping human-canine relationships. Such assumptions on love can mask systems of oppression, confinement, and exploitation of dogs. By exploring human-assistance dog interactions and reframing canine work as affective labor, this paper seeks to deepen our understanding of love’s complexities within interspecies relationships, broadening the concept beyond human-human connections and addressing its implications for assistance dogs.

While assistance dogs may empower individuals with disabilities to navigate daily life (Bennett and Goodall, 2024), it is essential to recognize the reciprocal nature of the relationship. How do the interactions between individuals with disabilities and their assistance dog companions create unique opportunities for connection and affective experiences, which differ from the relationships people have with their non-working dogs? What new affective patterns arise in the interdependent relationship between assistance dogs and people with disabilities, moving beyond human-centered concepts of independence? Exploring these inquiries has the potential to enhance the collaboration between disability studies and critical animal studies, thereby offering fresh perspectives on sociological investigations pertaining to emotions. This examination challenges the predominant anthropocentric beliefs in sociology and highlights the need to prioritize the physical and affective work of dogs (Section 3–4). By examining assumptions about love we can deepen our comprehension of assistance dogs and their caregivers, as this approach unveils the intricate and reciprocal emotional interactions between them (Section 5). One obstacle to such endeavors is the emphasis on independence over interdependence (Section 6). The unacknowledged canine work and affective experiences within a discourse of independence requires a critical perspective on speciesism – discrimination based on species membership, and how it intersects with ableism (Section 7). The relatively unexplored relationship between dogs and individuals with disabilities provides valuable insights for sociology, particularly given the global rise in demand for assistance dogs.

## 2 Sociology of emotions in more-than human worlds

Sociology maintains a deeply human-centric perspective, which reflects a speciesist bias, prioritizing the interests and welfare of humans over those of other animals, even as it acknowledges humans’ animal nature (Arluke, 2002; Nibert, 2003). The term “speciesism,” introduced by Ryder (1970, 1971), brought attention to this bias by drawing parallels between human treatment of animals and other forms of discrimination, such as racism and sexism. However, while Ryder’s concept of speciesism has sparked important ethical discussions, its sociological application lies in its capacity to critique the human-nonhuman divide that is embedded in institutional structures, everyday practices, and knowledge systems (Matsuoka and Sorenson, 2018).

“Speciesism does not refer simply to human relationships with other animals, but means socially, politically, economically, and culturally constructed everyday practices and a body of knowledge that supports such relationships. When Richard Ryder coined the term ‘speciesism’ in 1970, he discussed this as a form of prejudice and discrimination although he acknowledged that cruelties toward other animals are institutionalized” (Matsuoka and Sorenson, 2018, p. 1).

Speciesism reflects broader patterns of oppression and serves as a critical concept for sociological inquiry into social justice, prompting sociologists to reconsider how nonhuman animals are integrated into or excluded from societal structures, thus revealing new layers of inequality and bias. Historically, sociology’s human-centered definitions of society have largely excluded animals, even though classical sociologists like Max Weber recognized the potential for sociological study of animals (Weber, 1947; Peggs, 2012) with a few notable exceptions (Beirne, 1995; Nibert, 2013). This human-centered approach ties into the concept of human exceptionalism, the idea that humans’ rationality and symbolic capabilities make them fundamentally different from and superior to other animals (Dunlap, 1980).

In recent years, there has been growing recognition that nonhuman animals play a significant role in human society, and that many animals exhibit complex social behaviors, engage in intentional actions, participate in symbolic interactions, and have emotional capabilities (Taylor, 2011; Bekoff, 2007; Irvine, 2023). Especially within the last three decades, animals as sentient beings emerged as political actors with complex emotions, a topic explored in Anthrozoology, also known as Human-Animal Studies (HAS), which integrates perspectives from the social sciences, the humanities, and the natural sciences (Shapiro and DeMello, 2010). HAS researchers urge that nonhuman animals, whose agency has hitherto been ignored or compromised in anthropocentric narratives that uphold human exceptionalism, be viewed as “the latest beneficiaries of a democratizing tendency” in academic research (Ritvo, 2004). Thus, while sociological research primarily centers on humans, nonhuman animals “are so tightly woven into the fabric of society that it is difficult to imagine life without them” (Irvine, 2008, p. 1954). Therefore, it is crucial for sociology to embrace a broader perspective that transcends the conventional focus on humans and acknowledges the significance of nonhuman animals in society.

The relevance of animals in sociological research is further illuminated when considering the sociology of emotions. The field delves into the examination of how emotions are conceived, exhibited, and regulated within different social contexts since the 1970s (Hochschild, 1975; Kemper, 1978; Denzin, 1984). The sociology of emotions aims to explore how individual emotional experiences and expressions influence institutions, social norms, values, and interactions, as well as how these external factors reciprocally affect emotions. The last three decades saw remarkable progress within the field, and “the study of emotions is now one of the forefront areas of sociological inquiry” (Turner and Stets, 2012, p. 284), connecting micro and macro level of social reality. This paper does not aim to provide a comprehensive exploration of different conceptualizations of emotions and their distinctions from sensations, affects, moods, or sentiments. Nevertheless, it is clear that sociological studies on emotions have predominantly disregarded the intricate emotional experiences of animals and the affective dimension of human-animal interactions. Here, the limitations of human exceptionalism become more evident, as animals’ emotional lives and their capacity for symbolic interactions align with the core concerns of the sociology of emotions.

The absence of attention toward this subject can be attributed to various factors including methodological and ethical challenges, anthropocentric biases, the objectification of animals, institutionalized speciesism, and the dearth of interdisciplinary collaborations. Despite the recognition that animals possess feelings, sentiments, and emotions akin to humans, there has been a longstanding absence of comprehensive analyses on the human-animal bond and nonhuman emotions within the wider field of sociology. In 1979, Clifton Bryant critiqued sociology’s disregard of the “zoological connection” in understanding human behavior (Bryant, 1979, p. 399). Sociologists, he claimed, “have tended not to recognize, to overlook, to ignore, or to neglect (some critics might say deservedly so) the influence of animals, or their import for, our social behavior, our relationships with other humans, and the directions which our social enterprise often takes” (p. 399). He further suggested that the study of human emotions—so central to understanding social interactions—remains incomplete without considering how animals shape these emotional and social dynamics. Despite this call for attention lasting over four decades, and animals playing a significant role in social development, the interactions between humans and nonhuman animals, along with the complex social meanings they embody, have often been overlooked or marginalized in sociological research. Building on this critical gap, the following section examines the affective labor of assistance dogs, offering an opportunity to reconsider the idealized concept of unconditional love, which can obscure recognition of dogs’ physical and emotional work.

## 3 Affective labor and assistance dogs

Following Spinoza’s notion of affect, which involves the ability to both influence and be influenced simultaneously, this paper utilizes “affect” as a means to discuss pre-linguistic bodily sensations, moving past the customary terms of emotions, feelings, or sensations (Spinoza, 1994). Based on the examination of emotions, feelings, and sensations, affect theory delves into the complex interaction between bodily experiences and cognitive processes, shaping human perceptions, interactions, and expressions. This theoretical framework has undergone significant development, sparking discussions that demonstrate its intricacy and implications for comprehending the human condition (Stewart, 2007; Ahmed, 2004). While emotions are often regarded as being linguistic, affect theory considers pre-linguistic, non-verbal stimulations, feelings, and sensations, which can enhance sociological investigations on emotions and animals. This discussion has broadened its focus beyond human beings, leading to a notable exploration of animal affects (Bekoff, 2000). According to Donovan Schaefer, the affective perspective provides “a window onto the way that bodies operate prior to and in excess of language” (2017, p. 18). Affect theory is about:

“What makes bodies move, think, act and desire. In other words, affect theory is a theory of power, but a theory that sidesteps what I label the ‘linguistic fallacy’. The linguistic fallacy is a hidden presupposition sitting close to the heart of many projects in the humanities. It essentially says that in order to make things happen in the human world, a thought must be involved” (Schaefer, 2017, p. 19).

As a theory of power that transcends reason and thought, affect theory enhances our understanding of power dynamics in human-nonhuman interactions. It emphasizes the role of nonverbal communication and embodied experiences, particularly relevant to the interactions between assistance dogs and their handlers. The embodiment of affective experiences in dogs, as demonstrated by their ability to interpret emotional cues through body language challenges conventional models of affections that prioritize reason and verbal communication. This shift in focus encourages a more inclusive outlook on affective experiences and contributes to a deeper understanding of the various ways in which affect is expressed *within* and *across* species. Additionally, this critique of reason and emphasis on nonverbal communication resonates with disability studies, which also challenge normative standards of communication and cognition (Kafer, 2013).

The relationship between an assistance dog and a person with a disability operates through mutual affect, with each affecting and being affected by the other. This intricate emotional connection transcends mere functionality. Haraway (2008, p. 38), in discussing human-canine relationships, differentiates between companion animals and working animals based on “an economy of affection” and functionality, respectively. She suggests that affection poses a potential risk for animals, contrasting with the perceived safety of ethically bred working dogs. However, this oversimplification of the relationship between affect and functionality fails to capture the complex and meaningful bonds that form between assistance dogs and individuals with disabilities. Criticizing Haraway’s distinction between “pets” and working dogs based on skills and “an economy of affection,” Avigdor Edminster argues that separating affection from other economies is not feasible:

“While assistance dogs are clearly not solely dependent on ‘an economy of affection’ in the same way as a ‘pet’ might be, the various ways that the relationships between assistance dogs and clients are explained makes any clear distinction between ‘economies of affection’ and skillful work an uncertain proposition” (2011, p. 138)

The critique offered by Edminster challenges Haraway’s clear-cut distinction between pets and working dogs by emphasizing that it is impossible to fully separate affective bonds from functionality in the context of assistance dogs. In addition to their physical labor, assistance dogs also invest their affective wellbeing in their work by navigating complex social situations, processing sensory information, meeting the emotional needs of their handlers, and carrying out repetitive tasks. Assistance dogs are trained to carry out unique tasks that are beyond the capabilities of both humans and other animals (Arnold, 2011; Oliver, 2016). For instance, they have the ability to detect physiological changes in the human body and alert their handlers in a timely manner (Reeve et al., 2021). While guide dogs rely on visual cues to assist their handlers, medical-alert dogs rely on their keen sense of smell to perform effectively, establishing a crucial bond with their human partners (Reeve et al., 2021). These working dogs are not only highly skilled in their tasks but also deeply attuned to the emotional and nonverbal signals of their handlers, and can detect subtle changes in facial expressions, body language, hormone levels, and vocal tones (Mialet, 2020). While working, these dogs are not allowed to socialize with other humans or animals. This empathic understanding and affective responsiveness enable assistance dogs to provide comfort and enhance affect regulation among individuals with disabilities (Rodriguez et al., 2021). Their mere presence, companionship, and the release of oxytocin during interactions can lead to positive effects on mood, stress levels, and overall emotional health (Marshall-Pescini et al., 2019). Assistance dogs not only facilitate social interactions but also help in breaking down barriers, fostering social engagement, and reducing feelings of isolation for individuals with disabilities (McManus et al., 2021). This social dimension can influence affective experiences and contribute to a sense of belonging and identity for people with disabilities. However, the affective labor and wellbeing of these working dogs is overlooked in welfare discussions, which reflects “wider human exceptionalism” (Blattner et al., 2020, p. 5).

The concept of “emotional labor” introduced by Hochschild (1975, 2008) was groundbreaking in how it illuminated the invisible emotional management often required in certain gendered service and care professions. Hochschild distinguished “emotional labor,” specific to paid work, from “emotion work,” which refers to similar emotional management in unpaid context. Hochschild (1975, 2008) highlighted how individuals, especially women in traditionally “feminine” occupations like nursing, teaching, and service, manage their emotions as part of their professional obligations. This process involves not only the regulation of their own feelings but also the active facilitation of the emotional experiences of others, making emotional management an essential, though often underacknowledged, component of their work.

Although Hochschild initially focused on human experiences in gendered and commercial labor, this framework can also apply to assistance dogs. Kendra Coulter, use the term “emotion work” to describe how these dogs not only perform physical tasks but also manage their emotional states and help their human companions regulate their emotions. As Coulter notes, these working animals “are asked and expected to be in particular places and positions, to behave in specific ways, and to subvert their feelings or desires in order to meet the needs of people; that takes and is work, and provides yet another example of animals’ emotion work” (2016, p. 76). Additionally, they need to learn to ignore other animals while working to focus on their tasks diligently and act professionally by controlling their emotions.

The concept of emotional labor, as defined by Hochschild, remains widely used for analyzing interpersonal dynamics involving emotional regulation. Hardt and Negri, however, broadened this to “affective labor,” encompassing a wider range of relational activities beyond emotional regulation.

While emotional labor primarily focuses on the management of emotions in paid work contexts, affective labor “produces or manipulates affects,” which are prepersonal (Hardt and Negri, 2004, p. 108). This paper prefers the term “affective labor,” as it is better suited to address human-animal relations and nonhuman animal perspectives. The affective labor of assistance dogs exemplifies the intricate and expansive emotional regulation, display, and management that are central to the sociology of emotions, highlighting its complexity beyond the more limited concept of emotional labor. However, their affective care work seldom receives social recognition and it is a topic still underexamined (Coulter, 2016). As Coulter writes, “the study of multispecies work still comprises a very small proportion of the total collection of research in the sociology of work” (2016, p. 22).

This gap in recognition highlights the need for a multispecies perspective that critically examines the relationships between humans and nonhuman animals. Cary Wolfe, a prominent figure in animal studies and posthumanism, delves into the realm of affect theory to illuminate the complexities of these human-animal interactions (2010). By focusing on the affective intensities that surface during human-animal interactions, Wolfe highlights the nuanced emotions and sensations that transcend conventional modes of communication and cognition. Wolfe’s work invites a rethinking of anthropocentrism and opens up possibilities for more inclusive understandings of affect. Opposing “the fantasies of disembodiment and autonomy” (Wolfe, 2013, p. xv), Wolfe’s posthumanist discussion enables a more complex understanding of affective investments of humans and the taken-for-granted ways of experience. Wolfe’s examination of posthumanism prompts a critical reassessment of anthropocentrism by acknowledging the intricate affective connections that blur the boundaries between different species. Speaking of disability and service dogs, Wolfe writes:

“…instead of seeing nonhuman animal as merely a prop or tool for allowing the disabled to be mainstreamed into liberal society and its values, would not we do better to imagine this example as an irreducibly different and unique form of subjectivity– neither *Homo sapiens* nor *Canis familiaris*, neither “disabled” nor “normal,” but something else altogether, a shared trans-species being-in-the-world constituted by complex relations of trust, respect, dependence, and communication (as anyone who has ever trained—or relied on—a service dog would be the first to tell you)?” (Wolfe, 2013, p. 140–141).

Wolfe’s critique of the dualism between humans and animals aligns with the transformative nature of the affective labor performed by assistance dogs. The affective bond between an assistance dog and a person with a disability disrupts traditional distinctions between human and non-human experiences. This bond creates an opportunity to consider “interspecies solidarity,” which emphasizes respect, reciprocity, and the enhancement of working animals’ lives by acknowledging both their physical and affective labor (Coulter, 2020).

Building on the idea of attunement, Hélène Mialet provides further insights by focusing on diabetic alert dogs, describing them as loving, nonjudgmental “living prostheses” (Mialet, 2020, p. 2), capable of accessing “certain information about human individuality that humans themselves ignore” (2020, p. 3). For Mialet, dogs’ sense of smell and sensations make them ultimate ethnographers, reacting to miniscule changes in the body that are imperceptible to humans themselves. It is their affective capacity, responsiveness and acute sense of smell that make the dogs living prostheses (2020, p. 2). In addition to training, the establishment of a strong attunement and bond between the canine and their human companion is imperative for the success of this partnership. Mialet writes, “The trainer attunes to the dog, the dog attunes to the trainer; the dog attunes to the individual, the individual to the dog: all are ethnographers of each other, all inhabit each other worlds, all exchange properties” (2020, p. 7). While Mialet emphasizes attunement and the bond between dogs and their human counterparts, her portrayal may unintentionally promote an instrumentalist perspective that overlooks dogs’ affective labor and unique abilities, reducing them to mere extensions of the human body. This approach can undermine the dog’s agency and autonomy by suggesting they are solely functional in nature. It is important to acknowledge that while these dogs serve as empathetic companions, attuned to the emotional needs of their human partners, they also possess their own needs, desires, and capacities that extend beyond their utility to humans.

While Mialet highlights the importance of attunement, her framing of dogs as “prostheses” contrasts with other perspectives that emphasize their agency. For example, Vinciane Despret’s concept of “embodied empathy” offers a more reciprocal view of the human-dog relationship (2013).

This view contrasts with the idea of a “prosthesis,” recognizing the dog as an active participant who co-creates meaning and emotional bonds with their human counterpart, rather than merely responding to signals. Despret highlights the:

“feeling/seeing/thinking bodies that undo and redo each other, reciprocally though not symmetrically, as partial perspectives that attune themselves to each other… Empathy is not experiencing with one’s own body what the other experiences, but rather creating the possibilities of an embodied communication” (Despret, 2013, p. 51).

Highlighting the inseparability of affection and utility in the co-dependent relationships between assistance dogs and their human partners, this paper draws on Wolfe’s critiques of the species divide and liberal humanism to introduce fresh perspectives into the conversation surrounding assistance dogs. Liberal humanism often prioritizes human agency and rationality, which can marginalize nonhuman experiences and reinforce hierarchies between species. By highlighting the often-underestimated affective labor of assistance dogs, this investigation prompts a re-evaluation of these conventional hierarchies and dualisms in human-animal interactions. As Charlotte Blattner et al. (2020) observe, animal “labor has been a site of intense instrumentalization, exploitation, and degradation” (p. 4), yet they also emphasize animal agency “as a site of interspecies justice” (p. 6). Embracing the intricate affective interactions between humans and assistance dogs signifies a step toward a more comprehensive and empathetic understanding of affective encounters that transcend species boundaries. By combining affect theory’s emphasis on bodily interactions with the sociology of emotions’ focus on emotional management, we can gain a more nuanced understanding of the profound emotional and affective bonds formed between assistance dogs and their human companions. A crucial aspect of this endeavor involves exploring the concept of unconditional love attributed to dogs, which can obscure the physical and affective labor that assistance dogs perform—an issue that will be further explored in the following section.

## 4 Do assistance dogs love working for humans?

In the discourse surrounding assistance dogs, it is commonplace to assert that they love helping people. Organizations like Can Do Canines promote this idea, depicting assistance dogs as fulfilled by their work and enjoying intricate bonds with handlers (Assistance Dogs FAQs, 2024). Similarly, another organization named “Paws as Loving Support” underscores assumptions of unconditional love through their services. Moreover, financial donors to such assistance dog organizations often express sentiments affirming the deep bond between these animals and their human counterparts. One donor notes that an assistance dog’s capacity surpasses human limitations, that they never get bored and love their human companions unconditionally (Then Along Came Liberty, 2024). Rather than scientific rigor, anecdotal narratives about a vague notion of love determine the bond between a dog and a handler. If we accept that “the experience and expression of hardwired emotions is the product of learning” (Turner and Stets, 2012, p. 285), then reflecting on what love *does* rather than what love *is* within assistance dog literature, can contribute to improving canine welfare and critical work on the sociology of emotions. Rethinking “love” in assistance dog literature is essential for advancing human-canine interaction, as emotions are integral to forming and questioning social structures (Turner and Stets, 2012).

Despret (2013) argues that animals are active participants in their relationships with humans, and underscores the importance of adopting a more humble and curious stance when engaging with animals’ emotional lives. If “understanding an emotion means understanding the situation and social relation that produces it” (Bericat, 2016, p. 495), we must expand our perspective to acknowledge the full spectrum of affective states dogs may endure. What if assistance dogs are merely tolerating their job because they were not given any other chance since their birth into incarcerated spaces? As it is difficult, if not impossible, to fully understand what a dog needs, likes or wants, accounting for the best interests of all those involved in assistance dog partnerships necessitates a re-evaluation of love. Denying complex emotions to animals because it is difficult to study them directly does not eliminate the fact that animals experience a variety of emotions (Bekoff, 2000).

“Many emotions are wired into the body systems responsible for emotions, but their activation, expression, and use are highly constrained by the emotion culture of a society and the structure of those situations that call for individuals to experience and express particular emotions.” (Turner and Stets, 2012, p. 286).

It is outside the purview of this article to delve into the question of whether love can be classified as an emotion or simply a social bond. Nonetheless, “there is a conspicuous lack of serious reflection on the topic of love in the classical sociological tradition” (Rusu, 2017, p. 4). One of the reasons for that lack of involvement is that love is regarded as a private, psychological phenomenon. It is elusive and difficult to measure (Rusu, 2017). However, as Jackson (1993) puts it, “far from being just a personal, private phenomenon, love is very much a part of our public culture” (p. 202). Love, according to Jackson, is intertwined with the social and cultural setting in which individuals perceive it. It is a key element of the emotional background of social interactions, shaped by cultural, societal, and personal influences. Love is “characterized by its capacity to unite two individuals who are free to decide whether they want to be with each other in a shared sphere of intimacy” (Seebach, 2017, p. 54). In sociological examinations of this nature, the focal point of analysis lies in the evaluation and criticism of romantic, monogamous love and marriage. One notable instance is the emphasis on gender disparities, as highlighted by De Beauvoir (1972) when she stated “the word love has by no means the same meaning for both sexes” (p. 652). Building upon de Beauvoir’s perspective, the paper raises the question of whether the concept of love holds the same significance for both humans and dogs. Swen Seebach posits that “love can be criticized as a form of concealed discrimination and oppression” (2017, p. 62). Therefore, exploring the notion of unconditional love within the context of assistance dogs can offer a more nuanced analysis of the unequal power dynamics that love may serve to conceal.

In her work, Rudy (2011) writes that “emotional connection with real animals, connections based on love and shared lives, need to be included in the discourse of animal advocacy in order to maintain and model a better world for them” (2011, xii). Rudy explores the role of emotions in animal advocacy, arguing that love for animals can be “politicized” and used as the foundation for a broad animal ethic. She posits that “who we love is always a question of politics” (p. 25). Nevertheless, this article posits that an unexamined concept of love and affection can detrimentally affect the lives of assistance dogs. As Coulter writes, “the word love is a very political and significant metaphor and mobilizing force in animal communities and workplaces with many meanings and interpretations” (2016, p. 82). Therefore, when love is assumed without question, it may manifest as shallow, insincere, or even detrimental, neglecting to prioritize the genuine needs and welfare of the animals in question. Love can be “not really about caring for another,” but “a very self-centered emotion,” operating in a culture which values individualism and paternalism (Jackson, 1993, p. 210). Marran (2011) labels this form of assumed love directed toward and received from animals as “domesticating animal love” (p. 42). Domesticating love sees animals as things “onto which anthropomorphizing notions can be projected and through which social standards are maintained” (2011, p. 43). Examining the relationship between humans and animals through the lens of love could significantly enhance sociological investigations, given that this bond encompasses “many faces, some of which include moral elements, and some of which are fraught with moral dangers” (Gheaus, 2012, p. 589). The unchallenged assumptions such as “most companion animals love us nonjudgmentally” or “animal love lacks the control human beings have over their love and its expression” (Gheaus, 2012, p. 589) upholds oppressive social standards and anthropocentrism.

Martin Heidegger’s concept of “enframing” (*Gestell* in German) is a pivotal lens through which we can examine the ways in which love operates as an emotion glossing over power relations in human-assistance dog interactions. Enframing refers to a way of perceiving the world that reduces it to a resource to be controlled and optimized for human purposes (Heidegger, 1977, p. 12, 24). In the context of human-animal relationships, this lens can illuminate how assistance dogs are framed as tools to enhance human experiences and capabilities, particularly for individuals with disabilities. When applied to the use of assistance dogs, enframing suggests that these animals are seen as assistive technologies—resources designed to help individuals with disabilities navigate their environment more effectively. Heidegger’s concept of enframing is useful for understanding how assistance dogs might be viewed through a utilitarian lens. However, these dogs also resist this reductionist view by forming deep emotional connections with their handlers, offering companionship and care that go beyond their functional roles. This challenges the conventional view of enframing by introducing a more holistic way of understanding human-animal relationships—one that acknowledges the agency and affective contributions of the dogs themselves.

Viewing love as an enframing concept helps reveal how framing assistance dogs as merely “loving their work” risks neglecting their agency, individual needs, and complex affective experiences. The discourse of dog’s love for their work presents work for people as a core priority for dogs (Eisen, 2020). This concealment through “unconditional love” can lead to the invisibilization of the dogs’ complex affective landscapes and perpetuates anthropomorphism and human exceptionalism. This enframing through love might inadvertently simplify the relationship between assistance dogs and humans, reducing it to one of mere obedience and the fulfilment of human desires. As Seebach writes, “the danger of love and of the discourse of love rests in the projected possibility of creating a (homogeneous) one out of two, and to present such a (homogeneous) unity as something desirable” (2017, p. 63). In such unity, the affective experiences of assistance dogs, which go beyond utility and efficiency, can often be hidden from view. This notion aligns with Turner and Stets (2012), who assert that “whereas emotions operate to sustain or change social structural arrangements, it is equally true that social structures constrain the nature of emotional arousal” (p. 293). This perspective suggests that our understanding of love as expressed by assistance dogs may be shaped by conditioning and training, framed by human needs and expectations. Thus, the perceived emotional connection may reflect not only the genuine bond between humans and assistance dogs but also the influence of societal structures that dictate how such emotions are expressed and understood. Reflecting on the importance of sociological analyses on love to understand the society better, Seebach writes that “as a modern phenomenon,” love “had its role to play in the shaping of our current society, not just transporting inequalities of the past into the future, but reshaping the future by redefining the past” (2017, p. 75). Following this line of argument, we can say that love operates as a strong force within human-canine bond, which can cover over histories of selective breeding, reproductive control, practices of conditioning, intra-species isolation, coercion, and behavioral modification and training techniques, which are crucial to produce assistance dogs.

Assistance dogs are trained to perform specific tasks, and their behavior is modified with rewards or reinforcement (Audrestch et al., 2015). However, it is important to recognize that not all dogs successfully complete this training. Studies indicate that training failure rates can range from 50 to 70%, depending on various factors such as temperament, behavior, and health issues (Duffy and Serpell, 2012). As a result, many dogs are rehomed as pets rather than serving as assistance animals. If a dog fails to succeed in training, does this indicate a lack of desire or affection for the tasks, or does it reflect a mismatch between the dog’s natural temperament and the specific demands placed upon them? Framing assistance dogs as creatures that love their work may obscure the complexities of their emotional experiences and the coercive aspects of their training. The conditioning that assistance dogs undergo can create difficulties in distinguishing between genuine affection and learned responses.

Despite the impact of training on the expression of love in assistance dogs, some believe that it does not diminish the authenticity of the bond they form with their human partners (D’Souza et al., 2020). That is why it is crucial to adopt a more critical perspective on love within the context of the assistance dog-human relationship. This paper argues that love is a crucial factor in shaping human-assistance dog relationships, a dimension deserving closer examination. As closely intertwined with human social life, dogs provide a unique lens for investigating how emotions structure interspecies bonds, offering valuable contributions to sociological research on emotions. However, assumptions about canine love—such as the notion that dogs naturally love working for humans—risk obscuring the underlying systems of confinement and exploitation embedded in canine labor. Therefore, rather than focusing solely on dogs’ desire to please, it is important to examine the relationship through the lens of mutual respect, care, and affective reciprocity and an intersectional exploration of power. This perspective brings us to the concept of interdependence, framing human-assistance dog relationships as grounded in mutual care rather than in one-sided or purely functional interactions.

## 5 From independence to interdependence

Michalko (1999) reflections on his interactions with his guide dog, Smokie, offer an early exploration of interdependence in human-assistance dog partnerships. Unlike medical narratives that frame disability as mere impairment, Michalko regards blindness not as a deficiency but as an authentic way of being, enriched by his connection with Smokie. Where blindness is often perceived as a loss or limitation, Michalko reframes it as a unique mode of existence. His bond with Smokie enables him to reinterpret blindness, not as an inability, but as an experience shaped by emotional connection and trust (1999). This bond, emphasizing touch over the more distanced utility of a white cane, redefines blindness as something beyond a physiological difference and speaks to the deeper, affective dimensions of interdependence (Michalko, 1999).

Michalko’s challenge to ableist narratives that label blindness as a lack also resonates with Eva F. Kittay’s emphasis on dependency as an essential aspect of human life. Kittay underscores the importance of dependency in human life, and argues that “we cannot acknowledge our interdependency without first recognizing our dependency” (Kittay, 2015, p. 55). While dependency is inherent in human life, it has been historically associated with women, children, and individuals with disabilities, often leading to the infantilization and stigmatization, prompting individuals to pursue independence, which, according to Kittay, is a myth (Kittay, 2015). This stigma surrounding dependency negatively affects both disabled and nondisabled individuals’ sense of self-worth (Kittay, 2015, p. 58). Kittay writes,

“A consideration of dependency forces the question: can one still protect the benefits to be gained by disabled people’s demands for independence without re-stigmatizing those who do not benefit? Can we accept the inevitability of dependence without denying the negative effects of an *imposed* dependency on the lives of many disabled people? And can we accept reliance on dependency workers without subordinating their interests to those of the disabled person? (Kittay, 2015, p. 57).

This paper aligns with Kittay’s inquiries, considering assistance dogs as “dependency workers” whose labor often go unacknowledged within independence-focused discourses. Yet, as highlighted by Oliver (2016), Kittay’s feminist ethics of dependence is limited to interdependence between humans, overlooking the nuanced dynamics between humans and assistance dogs. While the narrative of unconditional love attributed to dogs can gloss over inequalities and obscure the labor and exploitation inherent in these relationships, emphasizing interdependence instead highlights the significant physical and affective labor performed by assistance dogs.

Unlike dependence, interdependence allows for the recognition of assistance dogs as active participants whose presence shapes their human partner’s lived experiences. Through physical tasks and affective attunement, assistance dogs play a crucial, skillful role, reshaping human experience beyond companionship. This understanding resonates with Sunaura Taylor’s framing of dependency “as an integral part of our world and relationships,” rather than negative or unnatural (Taylor, 2017, p. 210). For Taylor, all individuals live along “a spectrum of dependency” (2017, p. 210), which stands in opposition to liberal, ableist beliefs linking self-reliance with value. Recognizing interdependence fosters mutual respect, addressing the “dog’s existence as a separate being” with agency (Edminster, 2011, p. 133). Put differently, the narrative of independence reinforces a hierarchical dynamic that overlooks canine agency and the relational autonomy that exists between humans and dogs. Moving away from *independence* toward *interdependence* involves recognizing the shared dependency and vulnerability inherent in this relationship, where both humans and dogs contribute to each other’s wellbeing and development.

Wolfe’s emphasis on “a shared trans-species being-in-the-world” together with Kittay’s analysis of “dependency workers” and Taylor’s relational dependency challenge the notion of human independence which ignores the mutual co-dependency between assistance dogs and humans. Although dogs may not rely on humans for basic survival in the wild, their evolutionary history and selective breeding have fostered a deep interdependence with humans. This approach contrasts with human-centered notions of independence, which position animals as mere functional tools. Emphasizing interdependence highlights that dogs require care and respect just as much as their human companions. By recognizing this mutual dependency, the labor of assistance dogs challenges species bias, promoting a view of dogs as co-participants rather than instrumental aides. Thus, a shift toward interspecies interdependence not only contests speciesism but also advocates for respect for the affective states and wellbeing of assistance dogs. This perspective requires reconsidering speciesism and compulsory able-bodiedness, fostering a more inclusive attitude toward canine wellbeing. The subsequent section will delve into addressing and opposing speciesism as a means to restore a sense of interdependence.

## 6 Intersections of ableism and speciesism in the case of assistance dogs

The relationship between ableism and speciesism is essential for understanding the complexities of human-animal interactions, especially concerning assistance dogs (Taylor, 2017). These dogs enhance the autonomy and quality of life for individuals with disabilities (Rodriguez et al., 2021), by performing specific tasks while also offering companionship and emotional support, creating a bond that transcends utilitarian views. This dynamic challenges the traditional framing of assistance dogs solely as resources and calls for a nuanced understanding that recognizes their agency and emotional investment. Acknowledging both dogs’ physical and affective labor reframes the human-animal relationship as one of partnership, rather than utility, thereby contesting speciesism. However, prevailing speciesist attitudes often overshadow their contributions, fostering the idea that animals exist solely for human use, and neglecting their emotions wellbeing. Deeply ingrained in Western thought, speciesism perpetuates hierarchies that devalue nonhuman animals while simultaneously impacting individuals with disabilities. Exploring how these intersections shape perceptions and treatment of both individuals with disabilities and nonhuman animals provides valuable insights into the ethical implications of their relationships.

The emphasis on reclaiming humanity in disability studies and challenging hegemonic ideas of humanity in animal studies has presented difficulties in fostering coalitional politics between these two fields (Taylor, 2017). These tensions are further complicated by debates surrounding Peter Singer’s speciesism framework, which has been critiqued for its ableist underpinnings (Taylor, 2017). While my work engages with the critical examination of speciesism, I reject Singer’s utilitarian approach, which disregards the lived experiences of people with disabilities and perpetuates ableist comparisons between disabled individuals and animals by prioritizing reasoning and cognitive capacities (Singer, 1975). Instead, I advocate for a framework that recognizes the shared vulnerabilities and interdependencies between humans and nonhuman animals. Such a perspective aligns with Taylor’s argument that the oppression of animals and individuals with disabilities is deeply interconnected, suggesting that their paths toward liberation are intertwined (2017, p. xv). Taylor, writes:

“disability liberation cannot happen when our environments, the species who share those environments with us, and individual animals who live their lives entangled with ours continue to be seen through ableist and anthropocentric lenses that view them as things we humans can own and control–as discardable, fungible, and killable” (2017, p. 202).

Incorporating nonhumans into intersectional theory is essential for a comprehensive understanding of oppression. As Jackson (2020) emphasizes, this distinction is not solely rooted in biological differences; it is also deeply influenced by race and gender, contributing to processes of dehumanization and animalization. This racialized and gendered perspective intertwines with the concept of animality, weaving a complex web of abject humanity and racial hierarchies. Furthermore, as Taylor argues, “ableism is intimately entangled with speciesism” (2017, p. 57), highlighting the interconnectedness of these oppressive systems. This entanglement calls for a re-evaluation of how we treat both assistance dogs and individuals with disabilities. By recognizing the overlapping nature of these oppressions, we can advocate for a more inclusive approach that respects the rights and welfare of all beings involved.

The endeavor to restore humanity by individuals with disabilities who have endured historical dehumanization must not come at the cost of perpetuating animal oppression and speciesism. Practices such as selective breeding, favoring obedient traits, and applying standardized measurements to train assistance dogs can contribute to ableism by promoting conformity to normative standards that align with ableist expectations of utility and obedience. As many thinkers argue, “the oppression of [nonhuman animals] and speciesism overlap with other forms of oppression, such as racism, sexism, heterosexism, and so on” (Grauerholz et al., 2020, p. 131). The insufficiently theorized aspect of the assistance dog phenomenon presents a distinctive chance to advance intersectional analyses in sociological studies, particularly in the realms of disability, animality, and speciesism. Failing to address these interconnections “leaves wide gaps in our sociological understanding and theories of human society” (Grauerholz et al., 2020, p. 121).

The evolving field of Critical Animal Studies (CAS) encourages methodological and theoretical experimentation and calls attention to the interconnected systems of oppression that affect both humans and nonhuman animals (Matsuoka and Sorenson, 2018). Researchers were able to trace racial and social class interactions between people and animals in the context of European colonialism, for example, by concentrating on the history of dog breeding practices (Worboys et al., 2018; Wallen, 2017). Dogs in particular had a specific part in separating the ruling class from the general populace as well as the “civilized” from the “uncivilized.” In line with the affordances of a CAS perspective, there is a growing body of literature pertaining to the intersections of animality with race (Wallen, 2017; Scott, 2007), gender and sexuality (Sorenson, 2014; Stanescu, 2012), class (Worboys et al., 2018), colonialism (Montford and Taylor, 2020), biopower (Wolfe, 2013), and disability (Edminster, 2011; Taylor, 2017). Works that examine and challenge speciesism shed light on the interconnected origins of oppression and offer a thorough examination of its intersections with various social constructs. As Taylor asks, “if animal and disability oppression are entangled, might not that mean their paths of liberation are entangled as well?” (2017, p. xv). In this context, interdependence refers to a framework highlighting the mutual reliance and active contributions of humans and animals, moving the narrative away from the dog’s labor as a matter of mere obedience or affection. Hence, the examination of animals from a sociological perspective, the exploration of animals’ affective encounters, human-animal interactions, and the human and animal divide can offer significant insights into the complex intersections of disability, affect, speciesism, and animal welfare.

## 7 Discussion and conclusion

Integrating human-animal interactions and animal affect into current affect research and sociology of emotions broadens the scope of investigation beyond human experiences and contributes to a more nuanced and complex understanding of power relations. This expansion allows researchers to examine affective processes and expressions that might transcend species boundaries. Therefore, the utilization of sociological methods and concepts to investigate animals would contribute to a deeper comprehension of society, social interaction, the interconnected nature of oppression, and power relations (Stuart et al., 2013, p. 218). This intersectional perspective not only enriches sociological inquiry but also informs practices that promote equity and justice for both humans and nonhuman animals.

Acknowledging the affective labor of assistance dogs challenges anthropocentrism and fosters a more inclusive understanding of emotional engagement in human-animal relationships.

Drawing from Haraway (2008) insights on emotional labor, which “link feeling … to the issue of social justice” (p. 50), we can begin to unravel the preconceived hierarchy in human-animal interactions by recognizing the affective depth of dogs’ labor. Hochschild’s framework opens new avenues for examining animal labor, urging us to question our assumptions about assistance dogs’ unconditional love for their work. By linking this love to broader issues of power dynamics, abuse, and interdependence, we can better understand the full range of emotions these dogs may experience and what they “themselves seem to value most” (Eisen, 2020, p. 152). This perspective not only enhances their wellbeing but also encourages us to ensure that they are thriving in their roles rather than merely tolerating them.

Assistance dogs’ emotions, like excitement or stress, are often evident in subtle behaviors, making it vital to observe behavioral and physiological cues to better understand animal affect (Tomkins et al., 2011). By paying attention to behavioral cues, physiological responses, and cognitive assessments, a more comprehensive understanding of animal affect can be achieved. Research into animal affect should foster interdisciplinary collaboration across psychology, veterinary science, and animal behavior. Regular assessments by qualified trainers and veterinarians can help ensure that these dogs are emotionally healthy and capable of effectively assisting individuals. This revised perspective encompasses a more inclusive and empathetic comprehension of the affective experiences that bridge the species divide and challenge human exceptionalism. If emotions are social phenomena and dogs are part of our social life experiencing complex emotions themselves and with us, then sociology should integrate animals and human-animal interactions into its critical research. Researchers can develop a more intricate and thorough comprehension of the interconnected origins of oppression and power abuses by examining the impacts of nonhuman animals and their interactions with humans.

The intricate interdependence between an assistance dog and persons with disability necessitates a contemplation of care and a curiosity toward our interaction with dogs and addressing their welfare needs. Emphasizing assistance dogs solely as means of promoting human independence fosters a human-centric, speciesist view that overlooks canine experiences, values, and affect (Wadiwel, 2020; Oliver, 2016). Instead of perpetuating romanticized and misleading narratives of love and independence, it is essential to question assumptions, challenge potential abuses of power, and acknowledge the interdependence between humans and dogs. Embracing a deeper comprehension of love and emphasizing interdependence can cultivate relationships that are characterized by respect, communication, and dependency, thereby improving the welfare of both individuals with disabilities and assistance dogs.
